# Improving Image Quality by Accounting for Changes in Water Temperature during a Photoacoustic Tomography Scan

**DOI:** 10.1371/journal.pone.0045337

**Published:** 2012-10-11

**Authors:** Dominique Van de Sompel, Laura Sarah Sasportas, Anca Dragulescu-Andrasi, Sarah Bohndiek, Sanjiv Sam Gambhir

**Affiliations:** Molecular Imaging Program at Stanford (MIPS), Stanford University School of Medicine, Stanford University, Stanford, California, United States of America; The University of Chicago, United States of America

## Abstract

The emerging field of photoacoustic tomography is rapidly evolving with many new system designs and reconstruction algorithms being published. Many systems use water as a coupling medium between the scanned object and the ultrasound transducers. Prior to a scan, the water is heated to body temperature to enable small animal imaging. During the scan, the water heating system of some systems is switched off to minimize the risk of bubble formation, which leads to a gradual decrease in water temperature and hence the speed of sound. In this work, we use a commercially available scanner that follows this procedure, and show that a failure to model intra-scan temperature decreases as small as 1.5°C leads to image artifacts that may be difficult to distinguish from true structures, particularly in complex scenes. We then improve image quality by continuously monitoring the water temperature during the scan and applying variable speed of sound corrections in the image reconstruction algorithm. While upgrading to an air bubble-free heating pump and keeping it running during the scan could also solve the changing temperature problem, we show that a software correction for the temperature changes provides a cost-effective alternative to a hardware upgrade. The efficacy of the software corrections was shown to be consistent across objects of widely varying appearances, namely physical phantoms, ex vivo tissue, and in vivo mouse imaging. To the best of our knowledge, this is the first study to demonstrate the efficacy of modeling temporal variations in the speed of sound during photoacoustic scans, as opposed to spatial variations as focused on by previous studies. Since air bubbles pose a common problem in ultrasonic and photoacoustic imaging systems, our results will be useful to future small animal imaging studies that use scanners with similarly limited heating units.

## Introduction

Photoacoustic tomography (PAT) is a hybrid optical and ultrasound imaging technique that combines the high spatial resolution of ultrasound with the high optical contrast of diffuse optical imaging. It is a highly promising modality that has received increasing attention from the biomedical imaging community because of its ability to image in three dimensions, deep into living tissue. PAT is based on the illumination of an object with brief pulses of laser light. As the light diffuses into the tissue, it is absorbed differentially by the various regions of the tissue. The light absorption causes brief localized heating and an increase in pressure proportional to the amount of light energy absorbed. This leads to the generation of pressure waves that travel out of the tissue and can be measured by ultrasonic transducers. The detected signals are then reconstructed into an image of optical absorption using a computer algorithm.

Photoacoustic tomography systems of many different geometries have been proposed and investigated in the literature, such as circular [1]–[3], semi-cylindrical or curved [3]–[5], linear [6]–[8], planar [9]–[13] and hemispherical [14], [15] measurement apertures. This list is by no means exhaustive, and further references can be found in the recent review articles by Yao [16] and Beard [17]. A substantial number of photoacoustic tomography systems use water as a coupling medium to optimize transmission of the sound waves from the scanned object to the ultrasound transducers. This water is commonly heated to body temperature for small animal imaging, as is also the case for the imaging system used in this study, namely the Nexus 128 scanner produced by Endra Life Sciences (see the ‘Photoacoustic scanner’ section). Unfortunately, the water heating pump of this system sporadically injects air bubbles into the water. The manufacturer therefore recommends switching off the heating pump during a scan to minimize the risk of bubble formation on either the water-object or water-transducer interfaces. In this study, we found that the water tends to drop in temperature during scans of normal duration (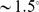
C during a 15 minute scan, typical of a single wavelength scan, and 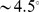
C during a 60 minute scan, typical of a multi-wavelength scan). In this work, we characterize the reconstruction artifacts that arise when ignoring these gradual temperature changes during the image reconstruction stage. In the Results section, we demonstrate the importance and efficacy of carefully monitoring the decreasing water temperature and applying software corrections to the changing speed of sound accordingly to optimize image quality. The improvements in image quality were shown to be consistent across objects of widely varying appearances, namely physical phantoms, an *ex vivo* mouse brain, as well as an *in vivo* subcutaneous tumor in a mouse. These results are significant because they show that a software correction for the temporal change in water temperature provides a cost-effective alternative to upgrading to a higher specification heating unit. Since air bubbles pose a common problem in ultrasonic and photoacoustic imaging systems, we believe that our results will help improve the quality of future small animal imaging studies that use scanners with similarly limited heating units.

Regarding the previous literature on speed of sound considerations in photoacoustic imaging, most studies so far have assumed a speed of sound distribution that does not change over the course of a scan, whether it be spatially homogeneous or heterogeneous [17]–[22]. Relatively little attention has been paid to the importance of monitoring water temperature over time, and continuously updating the speed of sound accordingly. There are some mentions of monitoring water temperature during a scan (eg. [5]), but no previous work has dedicated a study specifically to the characterization of artifacts generated by ignoring gradual changes in the speed of sound, as well as the efficacy of corresponding software corrections to improve tomographic image quality. The work presented in this paper addresses this gap. Note that we do assume a spatially homogenous sound distribution at any point in time in this paper. An investigation into the significance of modeling both spatial and temporal variations in the speed of sound is deferred to future work. However, since the water body is much larger than the heterogeneous volume inside the small animal being imaged, we expect that modeling temporal variations in the speed of sound will be more important than modeling spatial heterogeneities within the mouse. This is already indicated by the results in the Results section, which show a dramatic sharpening of in vivo images by correcting for the temporal temperature changes alone.

As for the remainder of this paper, the Methods section explains the methodology used for image reconstruction, the scan system setup, the dependence of the speed of sound on water temperature, the imaging subjects used, and the experimental procedures. The Results, Conclusions and Further Work sections present the results, draw conclusions, and give recommendations for future work, respectively.

## Methods

### Photoacoustic waves and spherical projection model

Photoacoustic wave propagation is described by the general wave equation, given by
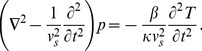
(1)where 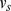 is the local speed of sound, 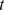 is time, 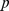 is pressure, 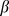 is the isobaric volume expansion coefficient, and 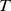 is temperature. The constant 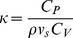, where 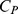 and 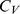 are the heat capacities are constant pressure and volume respectively, and 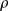 is the mass density. Using this model, and assuming that 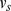 is constant everywhere, Wang et al. [23] showed that, for a particular transducer located at position 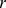,
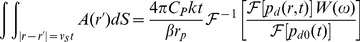
(2)where 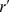 represents the coordinates anywhere in 3D space, 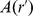 is the spatial absorption distribution, and 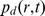 is the photoacoustic signal detected by the transducer. The operators 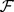 and 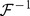 represent the Fourier transform and inverse Fourier transform, respectively. The signal 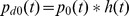 is the pressure recorded by the transducer after illuminating a point source, with a delay time of 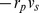. The signal 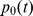 is the photoacoustic pressure from the point source with a delay time of 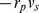, 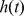 is the impulse response of the transducer, and 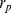 is the distance between the transducer and the point source. The constant 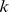 is defined by 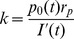, where 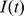 is the temporal illumination function, and 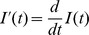. Lastly, 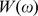 is a window function to band-limit the recorded pressure signals in order to prevent high frequency amplification by the deconvolution operation performed by the denominator 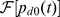. Eqn. 2 provides a spherical projection model, which we used as the basis for a filtered backprojection algorithm (FBP). All reconstructions shown in this paper were computed using the FBP reconstruction method.

### Photoacoustic scanner

**Figure 1:**
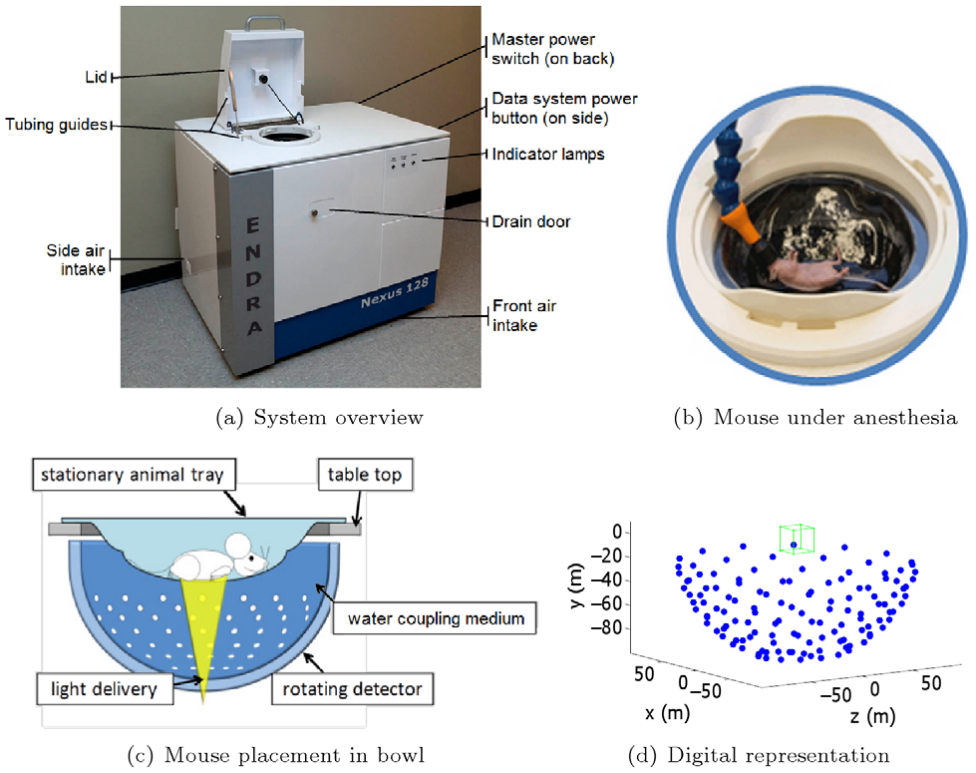
Nexus 128 scanner. (a–c) are reproduced with permission from http://www.endrainc.com. The green box delineates the spatial support of a representative reconstructed field of view. The dimensions of this reconstructed volume are typically set to 2×2×2 cm, and the isotropic reconstruction resolution typically to 0.2 mm.

In this study, we acquired photoacoustic scans using the Nexus 128 scanner, manufactured by Endra Life Sciences (Ann Arbor, MI, USA). Fig. 1 illustrates the system. The detection geometry consists of 128 unfocused transducers arranged on a hemispherical bowl. The radius of the bowl is 101.3 mm. A plastic tray is placed on top of the bowl for animal positioning. The tray is covered by a lid that must be closed while scanning (an interlock prevents scanning while the lid is open) as a protective measure to rule out the possibility of eye damage from the laser for the user. A webcam inside the lid allows users to monitor the mouse during a scan. The bowl is filled with water to provide acoustic coupling. A water heating system maintains the water temperature at 38°C between scans to enable small animal imaging. The readings of the water heating system's thermometer can be accessed in a log file. The water heating pump is switched off during scans to eliminate the risk of bubble formation. The blue dots in Fig. 1(d) indicate the transducer positions, and the green box indicates the spatial support of a standard reconstructed volume (100×100×100 with an isotropic resolution of 0.2 mm centered on the focal center of hemispherical bowl). The maximum field of view supported by the system is currently a sphere of radius 3.8 cm, again centered on the focal center of the hemispherical bowl. Each transducer has a circular detection surface with a diameter of 3 mm and records at a sampling rate of 20 MHz. The center frequency of the transducers is 5 MHz with a bandwidth of approximately 70%. To increase geometric sampling of the detection surface, the bowl is rotated in steps through multiple positions during a single scan. Usually, approximately 120 to 180 bowl positions, distributed uniformly over 360 degrees, suffice to obtain a high quality reconstructed image.

The system uses a tunable, near-infrared Nd:YAG laser that produces 7 ns pulses at up to 25 mJ per pulse. The wavelength can be tuned within a wavelength range of 680–980 nm. The repetition rate of the laser is 20 Hz. To reduce the effect of laser pulse power variations, and to increase the SNR of the recorded pressure signals, we average signals over a predetermined number of pulses (typically 100). At the laser repetition rate of 20 Hz, a standard scan using 180 bowl positions therefore takes 15 minutes per excitation wavelength.

### Dependence of speed of sound on water temperature

**Figure 2:**
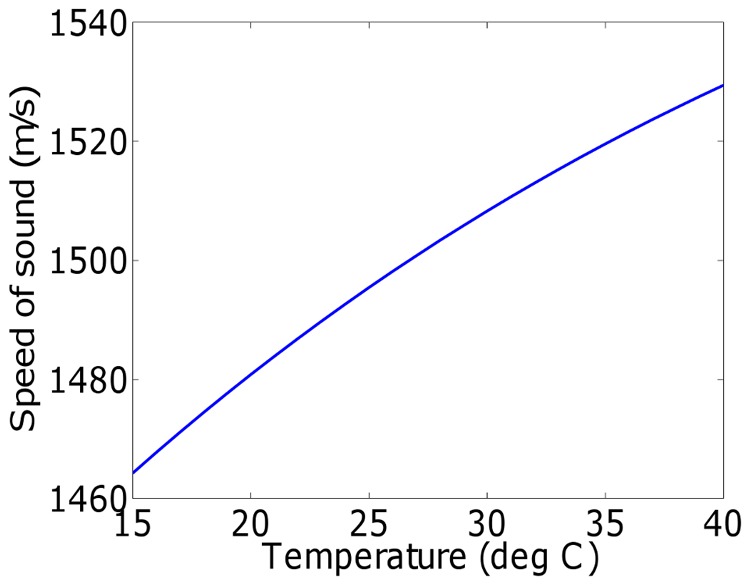
Speed of sound as a function of water temperature.

A number of studies have determined the speed of sound in water [24]–[28]. In this study, we use the nine-term Mackenzie equation [29], given by
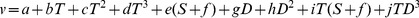
(3)where 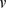 is the speed of sound in meters per second, 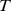 is the water temperature in degrees Celsius, 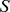 is the salinity in parts per thousand and 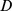 is the depth in meters. The coefficients of Eqn. 3 are given in Table 1, and a plot of the speed of sound as a function of temperature for 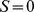 and 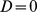 is given in Fig. 2.

**Table 1:** Coefficients for the polynomial modeling the speed of sound in Eqn. 3.

a	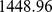	f	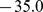
b	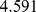	g	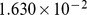
c	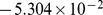	h	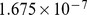
d	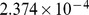	i	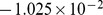
e	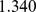	j	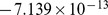

### Imaging subjects

#### Ethics statement

All animal work was conducted in accordance with the guidelines of and approved by the Administrative Panel on Laboratory Animal Care at Stanford University.

#### Subject preparation

**Figure 3:**
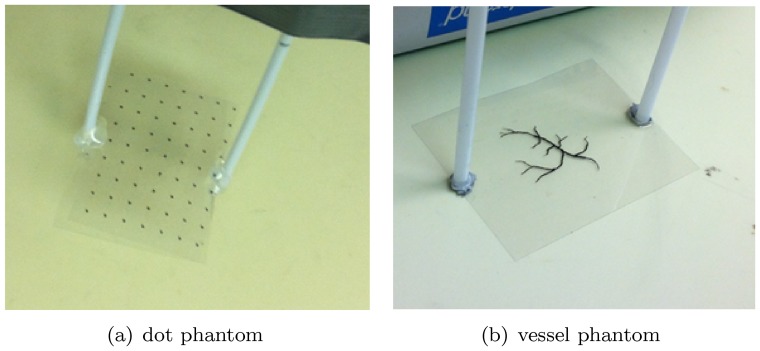
Phantoms. The vessel phantom in (b) is a print-out of a digital phantom provided in the k-Wave Toolbox [30].

**Figure 4:**
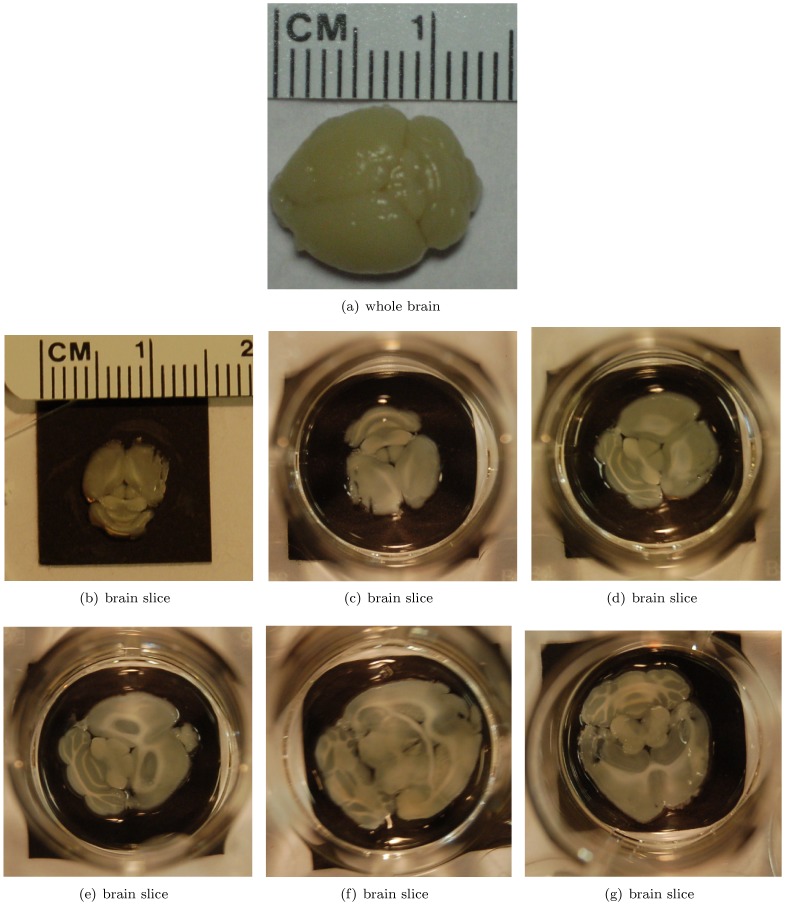
Perfused and excised mouse brain. (a) whole brain, (b–g) representative slices.

In this study, we have imaged two physical phantoms, an *in vivo* subcutaneous mouse tumor model, as well as a perfused and excised mouse brain. This allowed us to demonstrate our image improvement results for various realistic and practical circumstances. The phantoms were produced by printing a digital design onto a transparent overhead projector film using a standard black and white laser printer. Pictures of the printed phantoms are shown in Fig. 3, where the white straws used to lower the phantoms into the water are also visible. The *in vivo* subcutaneous mouse tumor model was obtained by subcutaneously implanting MDA-MB-213 breast cancer cells on the shoulder of a nude mouse. The mouse brain perfusion and excision was performed as follows. After placing the mouse under deep anesthesia, the animal's chest and abdomen were exposed by making a surgical cut along the midline. The frontal rib cage was cut, exposing the pericardium. A large gauge needle (18 gauge) connected to a syringe by a long PE tube was inserted into the left ventricle of the heart. The inferior vena cava was severed with scissors to allow the blood, saline and fixative to leave the body during perfusion. Using a syringe pump, the mouse was perfused with 20 mL of saline for 5–10 min followed by 20 mL of 10% formalin fixative solution. After perfusion, a craniotomy was performed and the brain was excised carefully and stored in 10% formalin. A picture of the excised mouse brain is shown in Fig. 4.

### Experimental procedure

In this study, we made use of three different scanning protocols:

1. protocol 1: 72 angles, 5 pulse averaging (scan time: 20 seconds)
2. protocol 2: 180 angles, 100 pulse averaging (scan time: 15 minutes)
3. protocol 3: 180 angles, 400 pulse averaging (scan time: 60 minutes)

All scans were acquired at a wavelength of 750 nm. Protocol 1 represents a typical preliminary scan used to verify that the object is positioned correctly. Protocols 2 and 3 represent realistic scanning protocols as well, where the duration of protocol 2 is typical of a single wavelength scan, and that of protocol 3 is typical of a multi-wavelength scan. The main difference of interest between the protocols is in their duration, leading to differing amounts of cooling of the water during the scan. Note that the different angle numbers and pulse averaging numbers result in additional sources of difference between the scans. However, the chosen parameters suggest that image quality should improve from protocol 1 to 3, as the signal sampling improves. In the Results section, however, we show that the increasing drop in temperature in fact outdoes these effects, and in fact results in an overall decline in image quality. Lastly, we used protocols 1 to 3 with the pump switched on as well as off. To differentiate between the different cases, we from hereon denote the scanning protocols with the pump switched off as protocols 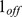, 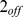 and 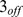. Those with the pump switched on are denoted as protocols 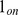, 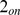 and 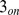.

## Results

### Printed phantoms

**Figure 5:**
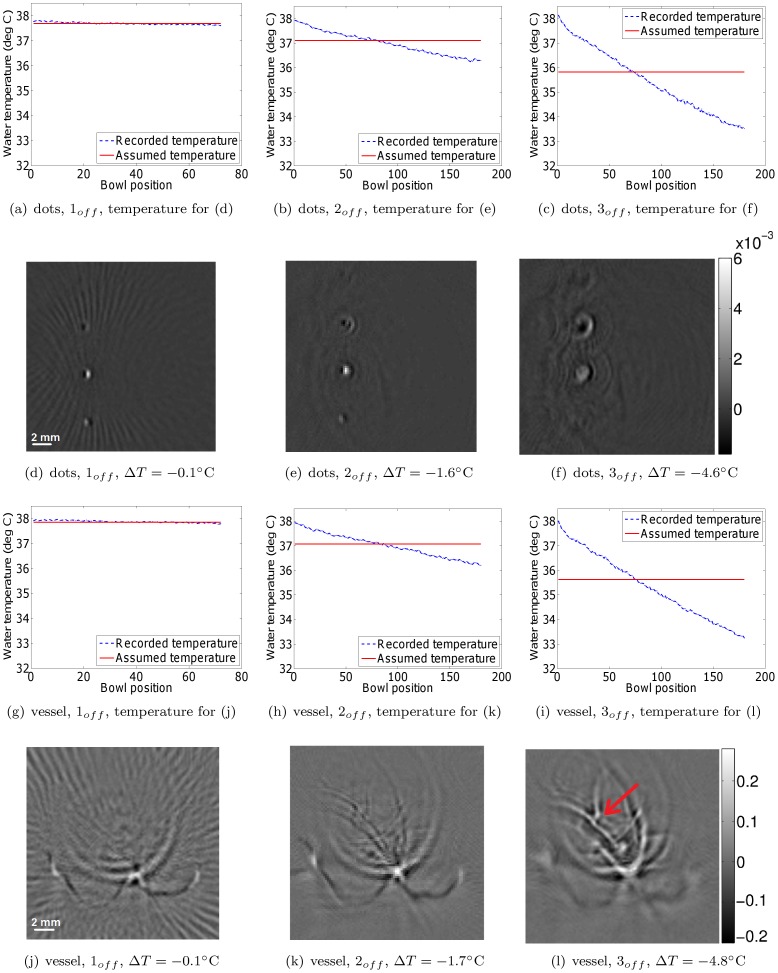
Dot and vessel phantom reconstructions from data acquired with the water heating system switched off. All reconstructions in a single row are displayed on the same grayscale (arbitrary units). 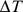 is the drop in water temperature during the scan. For reconstruction purposes, the water temperature was assumed to be the mean of the water temperature before and after the scan. The planes of the phantoms were not perfectly aligned with the horizontal planes of the reconstruction volumes. As a result, only parts of the phantoms are visible at a time. For example, only one column of dots is visible in the dot phantom, and only part of the vessel structures are visible in the vessel phantom. MIP images that show all structures are shown in Figs. 8 and 9. We show both slices and MIP images to illustrate the difference in appearance of the speed of sound artifacts in both types of images. The red arrow in (l) indicates artifact that is visually indistinguishable from true vessel structures.

As discussed in the ‘Photoacoustic scanner’ section, the standard protocol recommended by the manufacturer is to switch off the water heating pump during a scan to reduce the risk of air bubble formation. However, this causes the water temperature to drop slightly during a scan, and increasingly so for scans of increasing duration. As mentioned, we found this drop to be on the order of 1.5 degrees for a 15 minute scan, and 4.5 degrees for a 60 minute scan. While these temperature drops cause changes in the speed of sound in water of only 0.2% and 0.6%, respectively, we found that the impact on image quality can be significant. This is illustrated in Fig. 5, which shows reconstructed slices through the dot and vessel phantoms, acquired using protocols 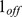, 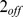 and 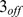. Also shown are the true (blue, dashed) and assumed (red, continuous) temperature histories.

For the dot phantom, the temperature drop results in a swirl-like artifact of increasing strength for longer scans. For the vessel phantom, the temperature drop results in the formation of extra vessel branches (see red arrow in Fig. 5(l)). Of critical importance here is that, unlike for the swirl artifacts, it is not visually obvious that these extra vessel branches are reconstruction artifacts. In other scans performed in our lab (not reported here), we noticed that this is a problem affecting any phantom containing a scene more complex than predictable dots, lines or squares. This last result in particular demonstrates that simply measuring the water temperature before and after a scan, and reconstructing by assuming the mean temperature value, is not sufficient.

**Figure 6:**
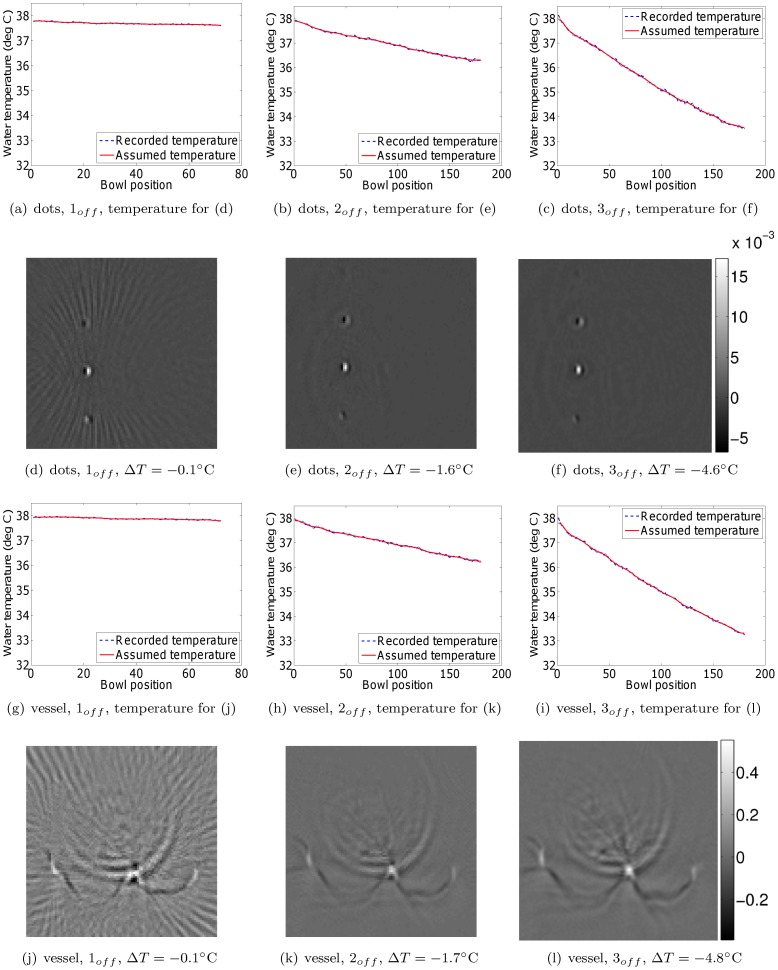
Dot phantom and vessel phantom reconstructions from data acquired with the water heating system switched off. All reconstructions in a single row are displayed on the same grayscale (arbitrary units). 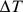 is the drop in water temperature during the scan. For reconstruction purposes, the water temperature was adjusted for each bowl position separately, based on the temperature record of the water heating system's thermometer.

**Figure 7:**
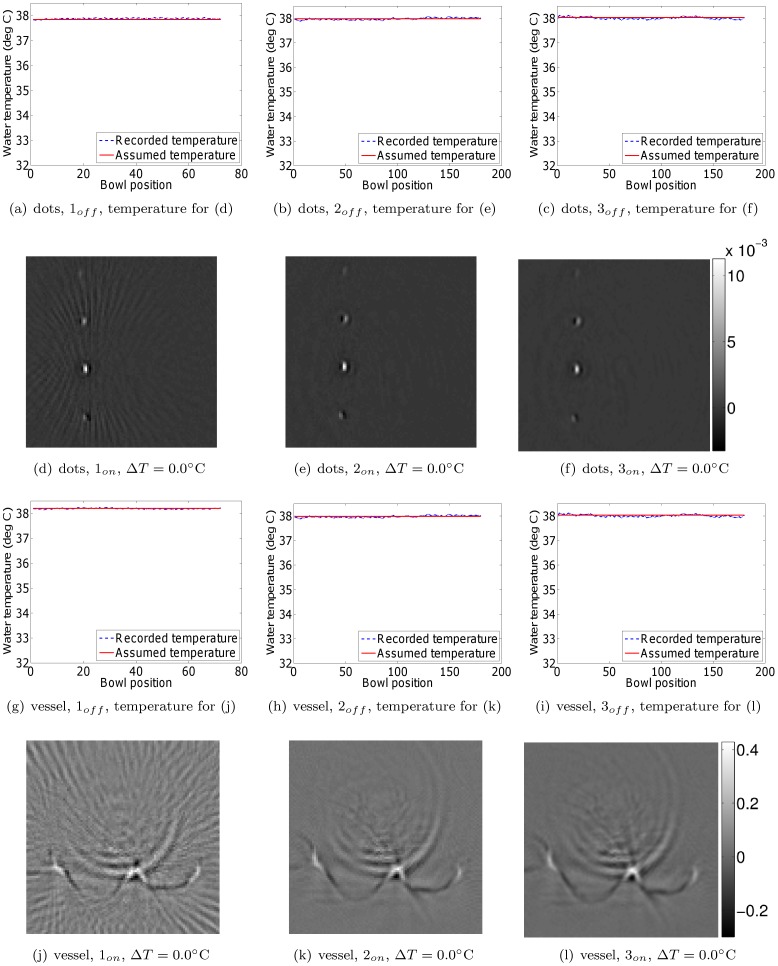
Dot and vessel phantom reconstructions from data acquired with the water heating system switched on. All reconstructions in a single row are displayed on the same grayscale (arbitrary units). During reconstruction, the water temperature was assumed to be the mean of the water temperature before and after the scan. Since the water pump was switched on, these temperatures were nearly identical and approximately equal to the target temperature of 38°C.

Next, we investigated the efficacy of monitoring the water temperature during a scan, and applying a post-acquisition temperature (and hence speed of sound) correction for each bowl position separately. This was done by reading the water temperature at the end of the signal acquisition at each bowl position. By way of validation, we compared the resulting reconstructions to those obtained by scanning the phantoms with the water heating system switched on and assuming a constant water temperature. The phantom positions were identical for every scan. Extra care was taken to make sure that leaving the water heating system on during the scan did not produce air bubbles. Figs. 6 and 7 show the resulting reconstructions for the scenarios of temperature correction and keeping the water pump on, respectively. To aid comparison, the figures have been placed in corresponding positions on successive pages, enabling rapid comparison using a pdf reader on a personal computer. The results demonstrate that applying a post-acquisition software correction to account for the changing water temperature results in an image quality equivalent to that obtained when the pump is kept running. The small differences are likely caused by a combination of temperature measurement inaccuracies and the potential motion of the overhead film due to water circulation in the case where the pump was on. Note also that the water temperature distribution within the cooling water bowl may not be perfectly uniform due to water convection. Regarding the issue of varying depth, we (1) assume that depth effects can be ignored due to the limited size of the bowl, and (2) point out that depth effects would affect the pump off and pump on scenarios equally. Lastly, notice that neither the temperature correction nor the continued operation of the water pump eliminated the out-of-plane artifacts visible in the reconstructions. These artifacts are due to the incomplete sampling of the measurement aperture, and are not the focus of this study. Nevertheless, the artifact reduction compared to the slices in Fig. 5 is clear.

**Figure 8:**
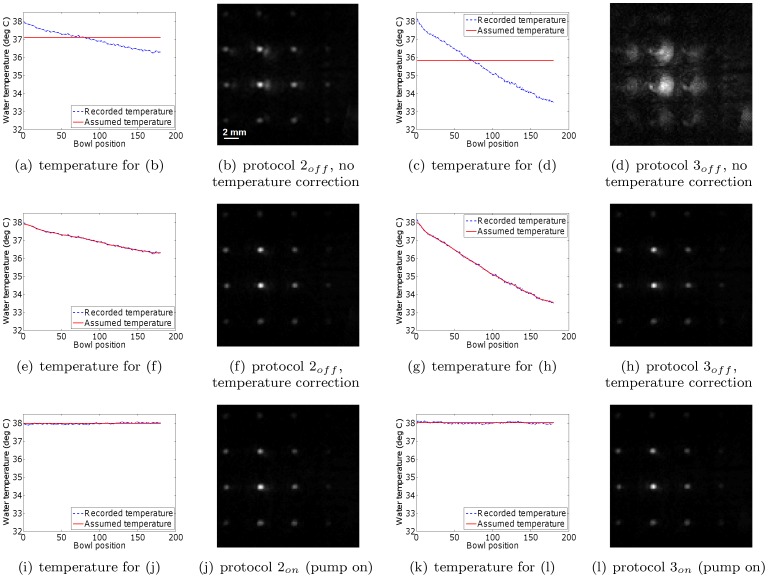
MIP projections of dot phantom reconstructions. All reconstructions in a single row are displayed on the same grayscale: 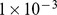 to 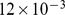 (arbitrary units).

**Figure 9:**
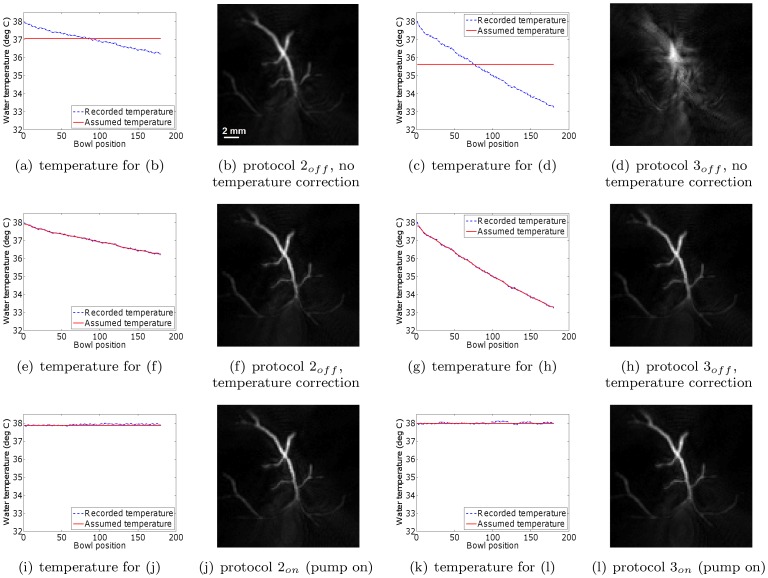
MIP projections of vessel phantom reconstructions. All reconstructions in a single row are displayed on the same grayscale: 0 to 1 (arbitrary units).

While viewing individual slices allows visualizing small local structures, they are not always useful for interpreting an entire 3D volume. For example, not all of the phantom structures are visible in the slices shown in Figs. 5, 6 and 7, since the phantoms happened to be positioned at a slight angle to the reconstructed planes. To circumvent this problem, one commonly looks at the maximum intensity projections (MIP) of reconstructions. We now examine how changes in the water temperature affect the appearance of MIP images. Since the temperature drop was not significant during protocol 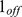, we from now show results for protocols 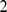 and 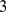 only, for the sake of conciseness. Figs. 8 and 9 show the maximum intensity projections of reconstructions of the dot and vessel phantoms. As before, not accounting for changes in water temperature results in a deterioration of the image that is increasingly more dramatic as the temperature drop increases. Note that the reconstruction artifacts in these MIP images take on the form of a blurring effect, which, fortunately, is less misleading than the artifacts seen in the individual slices in Figs. 5, 6 and 7.

### *In vivo* mouse tumor

**Figure 10:**
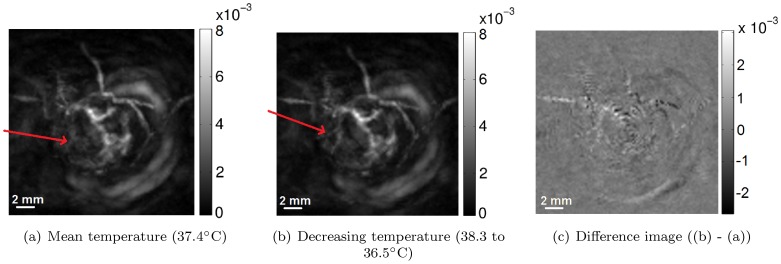
MIP projections showing the effect of correcting for decreasing temperature and hence decreasing speed of sound. By applying the speed of sound correction, the vessel bifurcation in the lower left quadrant becomes visible (red arrow). The vessel coming in from the top left also becomes clearer.

Here, we examined the benefit of temperature monitoring and subsequent intra-scan speed of sound corrections for an *in vivo* scan. Following the validations in the previous section with the pump switched on, we only compare reconstructions with the pump switched off, with and without post-acquisition temperature corrections. Fig. 10 shows reconstructed MIP images of a subcutaneously implanted MDA-MB-213 breast cancer cell line mouse tumor located on a mouse shoulder, scanned using protocol 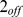. The red arrow shows that, by applying the speed of sound correction, the vessel bifurcation in the lower left quadrant becomes visible. Note also that the vessel coming in from the top left also becomes clearer as a result of the correction.

### Perfused and excised mouse brain

**Figure 11:**
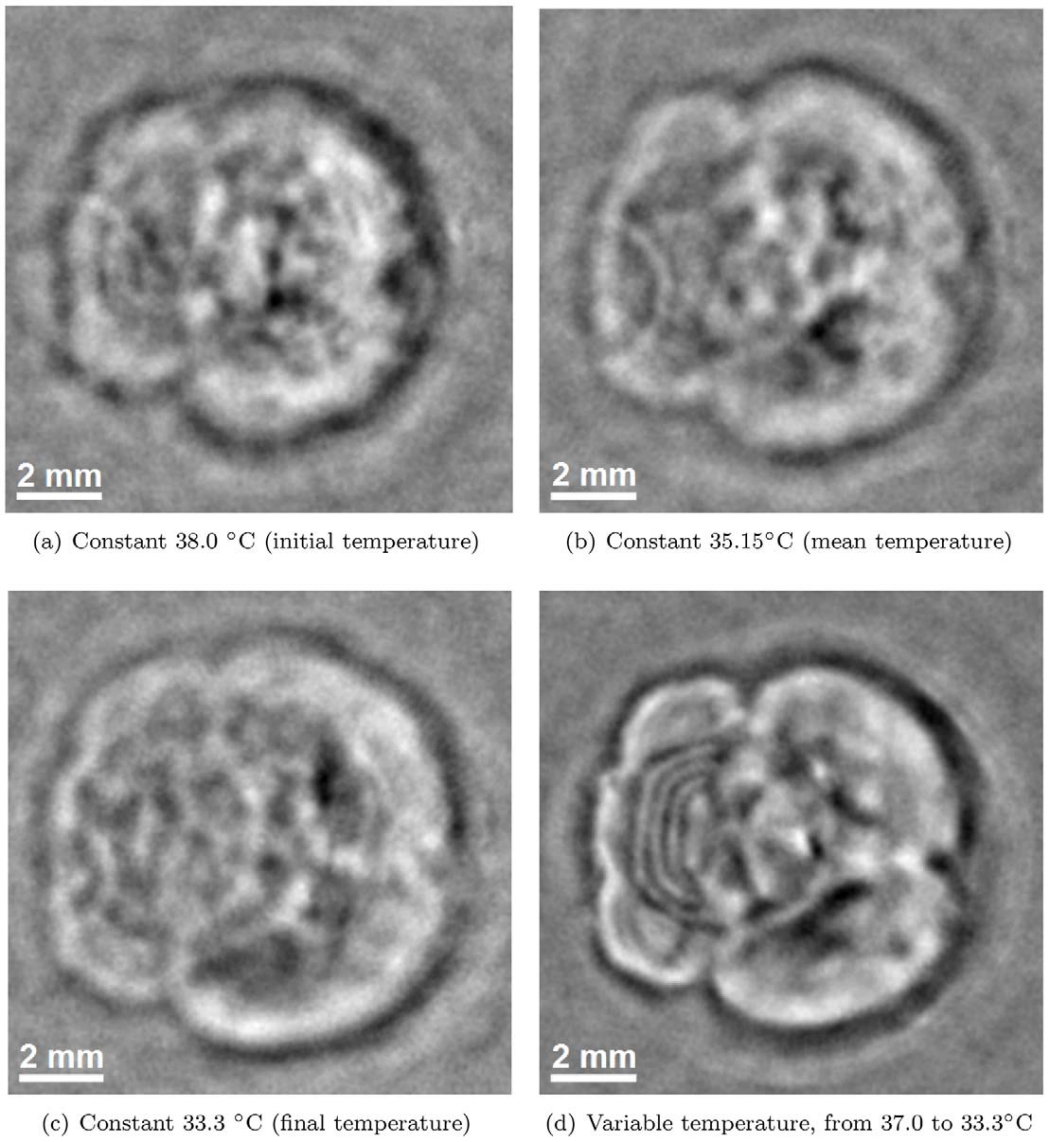
Slice through a reconstruction of a perfused and excised mouse brain. The correction for the changing speed of sound (d) greatly focuses the anatomical structures present in the image, compared to when reconstructing with the initial (a), average (b) or final (c) water temperature. These results illustrate that the image quality cannot be maximized by simply optimizing a single speed of sound value. Also note the close resemblance of (d) to the physical slice shown in Fig. 4(d). All reconstructions are displayed on the same grayscale: 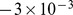 to 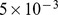 (arbitrary units).

Lastly, to demonstrate the benefit of variable speed of sound corrections on an image of yet a different appearance, Fig. 11 shows a reconstructed slice through a perfused and excised mouse brain (shown in Fig. 4). The improved focus of the anatomical features by accounting for the drop in the water temperature can be readily appreciated. These results clearly illustrate the main finding of this paper: the image quality cannot be maximized by choosing a single, optimal value for the speed of sound. Neither of the values corresponding to the initial, mean, and final measured water temperature achieved optimal results. Instead, the anatomical features were maximally focused only when the gradual temperature drop was fully modeled.

## Conclusions

The emerging field of photoacoustic tomography for molecular imaging is rapidly evolving with many new system designs and reconstruction algorithms being published. However, for imaging systems that use water heated to body temperature as a coupling medium, relatively little attention has been paid to the importance of water temperature changes during a scan. The work presented in this paper addresses this gap. For intra-scan temperature drops as small as 1.5 to 4.5 degrees (resulting in changes in the speed of sound of only 0.2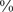 to 0.6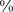 respectively), we found that image quality was significantly impacted unless the temperature change was explicitly accounted for. We showed that the image quality could be dramatically improved by continuously monitoring the water temperature and applying variable speed of sound corrections accordingly in the image reconstruction algorithm. The efficacy of such corrections was shown to be consistent across objects of widely varying appearances, namely physical phantoms, ex vivo tissue, and in vivo mouse imaging. It was also found that simply optimizing for a single speed of sound value could not achieve the same improvements in image quality. Next, temperature monitoring and post-acquisition speed of sound corrections were shown to give an image quality equivalent to that obtained when keeping the water heating system active during a scan, but without the increased risk of air bubble formation. Incidentally, it is true that incorporating a higher specification heating unit that does not introduce air bubbles into the water would solve the changing water temperature problem as well. However, for systems that do not already possess such a unit free of air bubbles, we have shown that software correction is a viable and accurate alternative to a hardware upgrade. This result will be useful to any research groups using scanners with similarly limited heating systems.

## Further Work

In this study, we assumed that the speed of sound was uniform throughout the image domain (encompassing both the coupling water and the scanned object) at any point in time. Further Work could investigate the importance of spatial heterogeneities in the temperature distribution within the water and/or the mouse at any given point in time. If found significant, such spatial variations could be modeled simultaneously with the temporal variations in a future algorithm. Lastly, we note that the underlying cause of the artifacts studied here (namely a gradual change in water temperature) is independent of the reconstruction algorithm used. However, the exact appearance of such artifacts may differ depending on the algorithm used. Further Work could characterize the appearance of changing speed of sound artifacts by algorithms other than filtered backprojection.
