# Correction: Improving Image Quality by Accounting for Changes in Water Temperature during a Photoacoustic Tomography Scan

**DOI:** 10.1371/annotation/fe1e40e1-5f1f-4ce2-9960-3d8849b83435

**Published:** 2013-05-14

**Authors:** Dominique Van de Sompel, Laura Sarah Sasportas, Anca Dragulescu-Andrasi, Sarah Bohndiek, Sanjiv Sam Gambhir

The Competing interests statement should read: Dr. Sanjiv Sam Gambhir serves on the board of Endra Inc., is a founding member, and has stock options. This does not alter the authors' adherence to all the PLOS ONE policies on sharing data and materials. 

